# Sex-Dependent Effects of the Histone Deacetylase Inhibitor, Sodium Valproate, on Reversal Learning After Developmental Arsenic Exposure

**DOI:** 10.3389/fgene.2018.00200

**Published:** 2018-06-15

**Authors:** Christina R. Steadman Tyler, Jane J. W. Smoake, Elizabeth R. Solomon, Estrella Villicana, Kevin K. Caldwell, Andrea M. Allan

**Affiliations:** ^1^Bioenergy and Biome Sciences, Los Alamos National Laboratory, Los Alamos, NM, United States; ^2^Department of Neurosciences, University of New Mexico Health Sciences Center, Albuquerque, NM, United States

**Keywords:** histone deacetylase inhibitors, prenatal arsenic, reversal learning, BDNF, valproate, cognitive flexibility

## Abstract

Several studies have demonstrated that exposure to arsenic in drinking water adversely affects brain development and cognitive function in adulthood. While the mechanism by which arsenic induces adverse neurological outcomes remains elusive, studies suggest a link between reduced levels of histone acetylation and impaired performance on a variety of behavioral tasks following arsenic exposure. Using our developmental arsenic exposure (DAE) paradigm, we have previously reported reduced histone acetylation and associated histone acetyltransferase enzyme expression in the frontal cortex of C57BL/6J adult male mice, with no changes observed in the female frontal cortex. In the present study, we sought to determine if DAE produced sex-dependent deficits in frontal cortical executive function using the Y-maze acquisition and reversal learning tasks, which are specific for assessing cognitive flexibility. Further, we tested whether the administration of valproic acid, a class I–IIa histone deacetylase inhibitor, was able to mitigate behavioral and biochemical changes resulting from DAE. As anticipated, DAE inhibited acquisition and reversal learning performance in adult male, but not female, mice. Valproate treatment for 2 weeks restored reversal performance in the male arsenic-exposed offspring, while not affecting female performance. Protein levels of HDACs 1, 2, and 5 were elevated following behavioral assessment but only in DAE male mice; restoration of appropriate HDAC levels occurred after valproate treatment and was concurrent with improved behavioral performance, particularly during reversal learning. Female frontal cortical levels of HDAC enzymes were not impacted by DAE or valproate treatment. Finally, mRNA expression levels of brain-derived neurotrophic factor, Bdnf, which has been implicated in the control of frontal cortical flexibility and is regulated by HDAC5, were elevated in DAE male mice and restored to normal levels following HDACi treatment. Levels of mRNA encoding glutamate receptor ionotropic NMDA type subunits, which have been linked to cognitive flexibility, were not related to the reversal learning deficit in the DAE mice and were not altered by HDACi treatments. These findings demonstrate that DAE alters frontal cortical HDAC levels and Bdnf expression in males, but not females, and that these molecular changes are associated with sex-dependent differences in cognitive flexibility in a reversal-learning task.

## Introduction

Over the past decade, the quality of our environment and the potential impact that it has on our biology, including cognitive development, function, and decline, has received much attention. Of particular interest is the presence of heavy metals, including arsenic, in our food and water sources. Exposure to this toxicant has been linked to a plethora of negative health outcomes ranging from cancer to heart disease to aberrant neurological development and function. Arsenic exposure occurs from a variety of sources, including occupational hazards (e.g., mining, manufacturing), direct consumption of food and beverages (e.g., rice, chicken, fruit juice, and wine) and water from wells (ASTSDR, 2011). The highest permissible concentration of arsenic is 10 parts per billion (ppb), as set by the World Health Organization (WHO) and United States Environmental Protection Agency (EPA). However, several regions around the world follow older guidelines of 50 ppb due to limited capability and infrastructure for improving water quality (Naujokas et al., 2013; US EPA, 2016; World Health Organization Arsenic Fact Sheet, 2016). Even in the United States, reports suggest that water obtained from at least 42.7% of private wells contains high concentrations of arsenic, comparable to countries such as Bangladesh, where researchers have extensively characterized the effects of arsenic poisoning on human health, particularly focusing on cancer (Chappells et al., 2014). Other epidemiological studies suggest that even low concentrations of arsenic, in the 10 ppb range, have negative impacts on human health, particularly cognition (Zierold et al., 2004). To this end, it is paramount to investigate the phenotypic effects of arsenic exposure, and to understand the basic biological mechanism of toxicity in order to develop and employ mitigation efforts.

Our lab, among others, has demonstrated a significant correlation between developmental exposure to lower levels of arsenic, in the 50 ppb range, and cognitive deficits (e.g., performance in learning and memory tasks), altered molecular mechanisms associated with stress responses, neurogenesis, cognition and depressive behaviors (Martinez et al., 2008; Tyler and Allan, 2013; Tyler et al., 2014). These findings correspond to epidemiological data suggesting a link between developmental or long-term arsenic exposure (ranging from 10 to 100 ppb) and significant deficits in cognitive capacity, including poor performance on indices of memory, attention, language, and executive function (Zierold et al., 2004; Rosado et al., 2007; Gong et al., 2011). Indeed, heavy metal toxicity has been widely reported to be associated with degenerative cognitive conditions, such as Alzheimer’s Disease (Squitti, 2012; Li et al., 2017). The mechanism of action for these effects is not well understood. While we have focused on arsenic’s influence on hippocampal circuitry, a limited number of studies have reported on its influence on frontal cortical circuitry and executive function (Martinez et al., 2011; Chandravanshi et al., 2014; Ashok et al., 2015). The NMDA subunit GluN2B, plays a significant role in executive functioning, and exposure to arsenic alters NMDA receptor subunit expression, particularly during development (Ramos-Chávez et al., 2015). Cortical deletion of *Grin2b*, the gene encoding GluN2B, impairs both choice learning and shift choice (reversal) behavior (Brigman et al., 2013), suggesting a connection between Grin2b and frontal cortex functionality. Our previous studies have shown that developmental arsenic exposure (DAE) affects epigenetic molecular signatures in the frontal cortex, particularly in adult male mice (Tyler et al., 2015a). Collectively, these results suggest that arsenic exposure may impair frontal cortex functioning perhaps via aberrant epigenetic signatures on important genes resulting in deficits in NMDA receptor expression.

The contribution of environmental influences, specifically toxins, on chromatin architecture, and ultimately gene expression, is currently under intense investigation. Pharmacological inhibition of epigenetic enzymes, particularly the use of histone deacetylate inhibitors (HDACi), results in enhanced cognitive outcomes in mouse models of neurodegenerative diseases (Graff et al., 2012; Graff and Tsai, 2013; Sanchez-Mut and Graff, 2015). Inhibition of HDACs allows for open, active chromatin configurations via inhibition of the removal of acetyl groups, which, along with methylation of DNA and histones, directly contributes to regulation of gene expression. It is well understood that alterations in chromatin architecture via epigenetic processes contribute to the mechanisms utilized in the mammalian brain for learning and memory (Graff et al., 2011; Zovkic et al., 2013). Medications that alter methylation or acetylation of histone proteins ultimately affect expression of genes, including brain derived neurotrophic factor (BDNF), that are crucial for synaptic plasticity and learning. Valproate, an anticonvulsive medication that also acts as mood stabilizer utilized for treatment of psychiatric conditions (e.g., bipolar disorder), has been shown to increase histone acetylation (Phiel et al., 2001). Studies suggest that it is valproate’s activity as an HDAC inhibitor that is the mechanistic basis for its actions in treating psychiatric disorders (Bredy et al., 2007; Bredy and Barad, 2008; Torrioli et al., 2010; Heinrichs et al., 2013; Wilson et al., 2014). Valproate administration alters BDNF expression that is concurrent with improvements in behavioral paradigms focused on assessment of learning and memory, primarily in the context of condition fear and extinction of conditioned fear (Bredy et al., 2007; Heinrichs et al., 2013). Our DAE paradigm induces changes in histone acetylation in the male mouse frontal cortex via altered histone deacetylate enzyme expression (Tyler et al., 2015a); thus, we hypothesize that the use of an HDACi, like valproate, may not only normalize aberrant histone acetylation in the brain but may also improve arsenic-induced cognitive deficits.

Recent interest in and awareness of differential effects of toxins and drugs in the female versus male brain has produced a mandate for research aimed at elucidating sex responses in many areas of biomedical sciences. Differences in *in utero* development, postnatal responses to external stimuli (e.g., pharmaceuticals, exercise, experience) and the subsequent development of psychiatric diseases have been identified in males and females (Barha et al., 2017; Cangemi et al., 2017; Gobinath et al., 2017). Epidemiological studies on the impact of arsenic on cognitive development in children demonstrate a significant sex difference, with females protected against arsenic’s effects (Rosado et al., 2007; Tyler and Allan, 2014). Indeed, we have observed a resilience to arsenic exposure in the female brain counter to findings in the male brain, especially with regard to epigenetic mechanisms of action (Allan et al., 2015; Tyler et al., 2015a, 2017). However, it is unclear if this arsenic-induced sex difference manifests as differences in behavioral flexibility, which, in part, underlies neurodegeneration. Based on our previous findings, we hypothesized that male mice exposed to 50 ppb arsenic during perinatal development would have impaired frontal cortex functionality. To test the validity of this assertion, we assessed performance in acquisition and reversal of the Y-maze learning task and the effect of valproate treatment on behavior in DAE male and female mice. We found that DAE males performed poorly on reversal learning tasks and that treatment with valproate throughout behavioral testing ameliorated these deficits. We also assessed HDAC protein levels, and Bdnf and Glutamate ionotropic receptor NMDA type subunits (Grin) mRNA expression to determine a potential mechanism of action for DAE-induced behavioral deficits and valproate responses. This research is the first to demonstrate that the use of an epigenetic modifier allows for reversal of arsenic-induced deficits in executive function.

## Materials and Methods

### Chemical Hazards

Arsenic is a human co-carcinogen and was handled with care in accordance with MSDS standards.

### Developmental Arsenic Exposure and Valproate Treatment

All animal procedures were approved by the Institutional Animal Care and Use Committee (IACUC) at the University of New Mexico and are in accordance with the NIH Guide for the Care and Use of Laboratory Animals, 8th Edition (National Academies Press, 2011). Female C57BL/6J mice (Jackson Laboratory, 55 days old) were assigned to either the control group, provided tap water (5 ppb arsenic), or the arsenic-drinking group, provided tap water containing 50 ppb sodium arsenite (Sigma-Aldrich) (Martinez et al., 2008). All animals were group housed (4 per cage), provided normal chow *ad libitum* (Teklad 2918, Harlan, Madison, WI, United States) and maintained in a temperature (22°C ± 2°C) and humidity (30–50%) controlled vivarium under a reverse light:dark cycle (lights off at 0800 h and on at 2000 h). After a 2-week acclimation period, females were introduced into male breeder cages and removed 4 days later. During the mating period, the male cage was provided the water treatment corresponding to that of the females to whom they were assigned. Following mating, females were singly housed during gestation and continued consumption of assigned water. Offspring were weaned on postnatal day 25 and placed on standard tap water and chow. At 70 days of age, offspring from control and arsenic-drinking dams were assigned to either vehicle or valproate treatment groups. To ensure 85% free feeding body weight, necessary for appropriate behavioral training, offspring were food restricted for approximately 2 weeks. During this time and subsequent behavioral training and testing, animals were administered a daily I.P. injection of either 50 mg valproic acid/kg body weight (Sigma-Aldrich) in 0.3 ml or sterile saline. Injections were given in the afternoon following the end of the behavioral assessments prior to returning the mice to the vivarium. Each animal received an injection of valproate for the entirety of the acquisition and reversal behavioral assessments. The duration of this treatment period was directly dependent on successful completion of behavioral tasks.

### Behavioral Assessment of Frontal Cortical Function

Assessment of executive function in rodents is limited to cognitive flexibility and response inhibition using attentional set shifting behavioral assays. The Y-maze forced alternation task was used to determine the effects of DAE and valproate treatment on frontal cortical function and compulsive/impulsive behavior through testing of working memory and response to stimuli. The Y-maze consisted of a clear Plexiglas hallway (76 cm) with the two arms (35 cm each) serving as two separate chambers consistently paired with visual and odor cues. The right arm contained a brown circular cue located terminally and a cinnamon odor provided medially under perforation in the maze arm floor. The left arm contained a black curved arrow cue paired with a garlic odor cue in the same locations. Eleven animals from different litters per sex, per drug treatment, and per developmental exposure were trained and tested. Animals were randomly assigned to either cinnamon-circle (CN) cues (right side) or the garlic-arrow (GR) cues (left side). Testing occurred in two sessions during the diurnal cycle at 0830 and 1400 h with each session consisting of three trials per animal. The maze was rotated 180° between sessions. Animals were habituated to the maze for 10 min with no cues present 1 day prior to training. For testing, the animal was allowed up to 4 min to make a choice after being released at the base of the Y-maze; on average, most animals made an arm selection within 45 s. Animals that traveled to the end of the assigned arm (CN or GR) received a “correct” score for the trial and received a food reward (small pellet of chow). Any other behavior, including choosing the incorrect arm, initial traveling down the correct arm but not obtaining the reward or failure to choose an arm during the allotted time was designated as an “incorrect” score. Successful acquisition of the Y-maze was defined as an animal’s choosing the correct arm 78% of the time within three consecutive sessions (7 out of 9 trials). Our criterion was based on studies performed by Shoji et al. (2012). In these behavioral paradigms, animals were trained daily until they reached an average of 80% correct responses. Once acquisition criterion was achieved, the cues associated with reward were switched to assess reversal learning. Therefore, animals initially rewarded for choosing the CN arm (right side) were subsequently rewarded for choosing the GR arm (left side). To obtain criteria during the reversal phase, animals had to choose the newly rewarded arm correctly 78% of the time within three consecutive sessions (7 out of 9 trials). Approximately 14 days was required for both initial acquisition of the task and subsequent reversal training. All animals acquired the initial task but those that failed to successfully meet reversal criterion were given a maximum score of 84. All 42 animals were tested through trial 1 before assessment of trial 2 (i.e., animals were not given trials 1–3 sequentially). The time between the two trials ranged from 30 min to 2 h, depending on the level of learning. The Y-maze was cleaned with 70% isopropyl alcohol between each animal. Upon obtaining reversal criterion, each animal was euthanized the following morning; brains were removed, dissected into appropriate regions, snap frozen in liquid nitrogen, and stored at -80°C.

### Assessment of mRNA Expression Using Quantitative PCR

mRNA expression levels for Bdnf exon IV and exon VI were assessed in frontal cortex tissue using previously published methods (Caldwell et al., 2008). Tissue was collected within 15–17 h after the last afternoon session following obtaining reversal criteria. Briefly, total RNA, including microRNA, was purified with the Ambion mirVana^TM^ miRNA isolation kit (cat: A1561, Life Technologies) following the manufacturer’s protocol with slight modification. Tissue homogenate in RLT Plus buffer from the previous gDNA and mRNA co-purification was substituted for tissue lysis with the buffer provided in the mirVana miRNA isolation kit. The standard procedure resumed with the addition of miRNA homogenate additive to the tissue homogenate. Total RNA was quantified on a NanoDrop 1000 (260/280 > 1.9) and stored at -80°C. In compliance with MIQE standards, melt curves were analyzed to verify a single target was amplified, no template and reverse transcription negative controls were included on every qPCR plate, and only *C*_T_ values below 35 cycles were used for analysis (Bustin et al., 2009). Endogenous expression of hypoxanthine phosphoribosyltransferase (Hprt) served as the reference gene for mRNA analysis. Hprt expression was not altered by DAE nor by valproate treatment. Gene target fold expression values were obtained using the comparative CT method (Livak and Schmittgen, 2001), and statistical analysis was conducted on GraphPad Prism 6 Software, version 6.03 (GraphPad Software, San Diego, CA, United States). Primer efficiencies for all variants of the Grin and Bdnf transcripts were between 0.986–1.0 and 0.90–1.0, respectively. Primers are presented in the **Table 1** below.

**Table 1:** Listing of the primer sequences for mRNA analyses.

Target	Primer	Sequence	Amplicon
Bdnf Total	Bdnf FWD mRNA	TCA TAC TTC GGT TGC ATG AAG G	123 bp
	Bdnf REV mRNA	AGA CCT CTC GAA CCT GCC C	
Bdnf Var3 (Exon 4)	Bdnf Var3 FWD1	CAG AGC AGC TGC CTT GAT	159 bp
	Bdnf Var3 REV1	GCC TTG TCC GTG GAC G	
Bdnf Var4 (Exon 6)	Bdnf V4-1 FWD	GTG ACA ACA ATG TGA CTC CAC T	85 bp
	Bdnf V4-1 REV	ATG GTC ATC ACT CTT CTC ACC TG	
Grin1 (Nmda1)	Grin1-1 FWD	GCC CGA CCC TAA AAA GAA AG	92 bp
	Grin1-1 REV	TGC TCG TGT CTT TGG AGG AC	
Grin2a (Nmda2a)	Grin2A FWD	ACG TGA CAG AAC GCG AAC TT	173 bp
	Grin2A REV	ATC TCC AAA CAC CAA GCC AT	
Grin2b (Nmda2b)	Grin2B FWD	GCC ATG AAC GAG ACT GAC CC	190 bp
	Grin2B REV	CCA TTA TCA TAG ATG AGC CC	


### Assessment of Protein Levels Using Immunoblotting

Assessment of protein levels was performed using previously established immunoblotting protocols (Tyler et al., 2015a). Frontal cortex tissue lysates from male and female offspring exposed to arsenic (or control) and treated with valproate (or saline) were prepared from tissue collected within 15–17 h of the afternoon session following obtaining reversal criteria; the nuclear fraction was isolated for protein analysis (Buckley and Caldwell, 2003; Caldwell et al., 2015). Antibody concentrations and total protein quantity (15 μg) were optimized to fit the linear range of signal detection. Immunoblot transfer was performed using a Mini Blot Module (cat: B1000, Life Technologies) onto an Immobilon-FL Transfer Membrane. Blots were incubated in LI-COR Blocking Buffer (cat: 927-40000, LI-COR) overnight at 4°C and subsequently incubated with anti-HDAC1 (Active Motif, #40967) or HDAC5 antibody (Novus, #NBP2-22152) diluted 1:1,000 in PBS-T for 3 h at room temperature (RT). IRDye 680 Donkey anti-Rabbit IgG secondary antibody (cat: 926-68073) was applied diluted 1:10,000 in PBS-T for 45 min at RT without light exposure. Imaging of the immunoblots was conducted using a two-channel Odyssey Infrared Imaging System (cat: LIC-8201-00, LI-COR) and quantified using Image Studio version 5.0 (LI-COR Biosciences). Protein detected by immunofluorescence was corrected to the total protein as quantified by Coomassie staining.

### Statistical Analysis

ANOVA was conducted on behavior, protein and mRNA data using SPSS (IBM v.24) and GraphPad Prism (version 6.03, GraphPad Software, San Diego, CA, United States) software. Assessment of the impact of arsenic exposure and subsequent HDACi treatment on behavior was conducted using a three-way ANOVA. Protein and mRNA data were analyzed as a 2-factor ANOVA; measurements were taken for one sex per assay. Bonferroni corrected *post hoc* (*t*-test) analyses were conducted where indicated. The number of samples per condition for each assay is presented in the results section and figure legends.

## Results

### Valproate Treatment Ameliorates Deficits in a Sex- and Exposure-Dependent Manner

**FIGURE 1:**
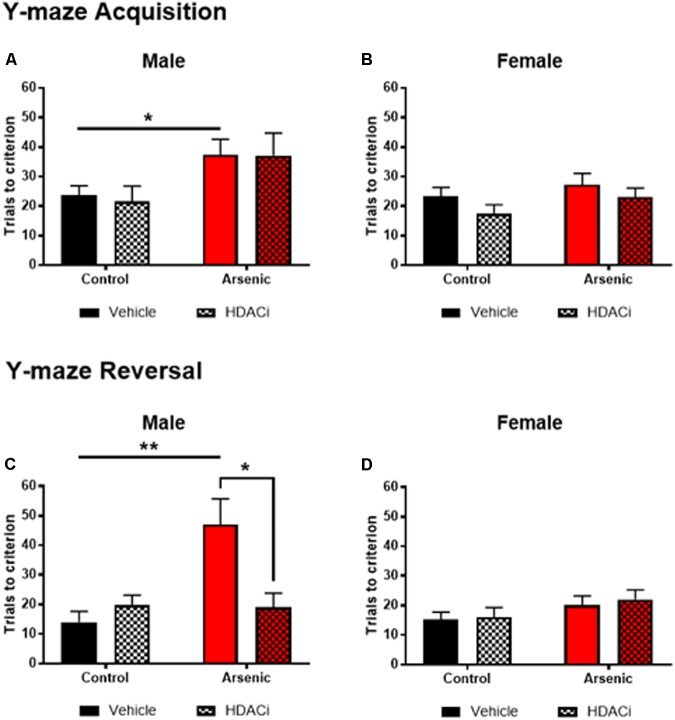
Trials to reach criterion in Y-maze acquisition (top row) and during reversal (bottom row) for male **(A,C)** and female **(B,D)** control (black bars) and arsenic-exposed (red bars) animals treated with vehicle (solid bars) or HDACi (valproate, stippled bars) every day during the training and testing. Data are presented as mean ± SEM, *n* = 8–10 per group and condition. ^∗^*p* < 0.05, ^∗∗^*p* < 0.005. Statistical details are provided in the “Results” section.

To determine the impact of DAE on frontal cortex functioning and behavioral flexibility, animals were trained on acquisition and reversal criteria using the Y-maze. In addition, the effects of valproate on acquisition and reversal learning in control and DAE mice were assessed. Three factor ANOVA (SPSS v 24) was utilized to analyze the effect of DAE, treatment with valproate (HDACi), and sex on behavior. Data presented in **Figures 1A–D** demonstrate a significant main effect of DAE on initial acquisition, *F*(1,58) = 10.68, *p* = 0.002, and reversal performance, *F*(1,58) = 8.70, *p* = 0.005. A significant main effect of sex was observed for acquisition performance, *F*(1,58) = 5.19, *p* = 0.026, with a poorer acquisition performance among male, arsenic-exposed, vehicle treated mice (*p* = 0.05), **Figure 1A**. This group also had significantly impaired performance during the reversal phase of the task (*p* = 0.005), **Figure 1C**. Performance deficits were not evident among female, arsenic-exposed, vehicle treated mice. Differential performance was observed on reversal criteria, with a significant interaction between DAE and valproate (HDACi) treatment, *F*(1,58) = 6.32, *p* = 0.015. Male, arsenic-exposed, valproate-treated animals performed significantly better during reversal training than the vehicle-treated counterparts (*p* = 0.024), though no differences among VPA-treated nor vehicle-treated control males was detected (**Figure 1C**). Reversal performance was also significantly impacted by valproate treatment and sex, with a significant interaction among all three variables, *F*(1,58) = 6.4, *p* = 0.014. Potentially, this interaction is caused by lack of deficit on reversal performance among female, arsenic-exposed animals, in direct contrast to the behavior deficit in arsenic-exposed males that was ameliorated by valproate treatment (*p* = 0.025). DAE produced significant behavioral deficits in male performance during acquisition, *p* = 0.05, and reversal training, *p* = 0.005, including an arsenic exposure by HDACI treatment during reversal learning, *p* = 0.003. For female animals, there were no significant effects of developmental exposure or valproate treatment, for either acquisition or reversal performance in the Y-maze. Valproate treatment improved performance on the reversal task for only the males.

### Treatment With Valproate Reduces HDAC Levels in Males

**FIGURE 2:**
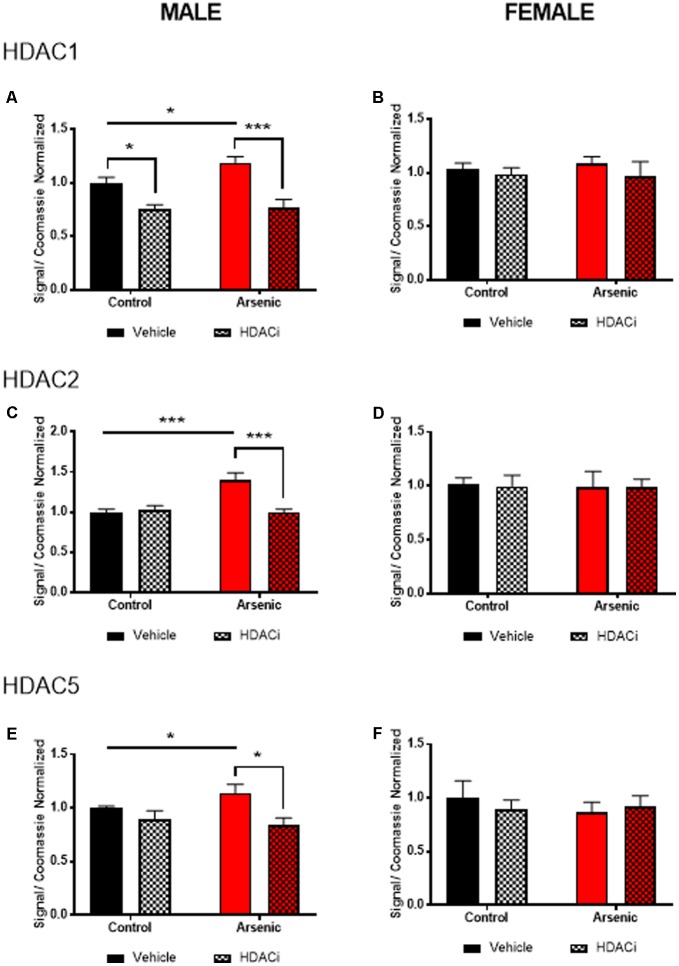
Levels of HDAC1 **(A,B)**, HDAC2 **(C,D)**, and HDAC5 **(E,F)** assessed by immunoblot using the nuclear fraction of the frontal cortex from male **(A,C,E)** and female **(B,D,F)** control (black bars) and arsenic-exposed (red bars) animals treated with vehicle (solid bars) or HDACi (valproate, stippled bars). Data are mean ± SEM, *n* = 6–7 of immunoblot signal corrected to total protein (Coomassie stain) and expressed relative to control vehicle condition. ^∗^*p* < 0.05, ^∗∗∗^*p* < 0.001. Statistical details are provided in the “Results” section.

To determine the effect of arsenic exposure and valproate treatment on HDAC levels, we assessed levels of HDAC enzymes 1, 2, and 5 in the frontal cortex (nuclear fraction), **Figure 2**. Tissue was prepared approximately 15–17 h following the last afternoon session after achieving the Y-maze reversal criteria; thus, all subjects experienced 14 days of behavioral activation prior to protein quantification. No significant main effects for DAE were found for HDAC1 or HDAC5 for males; however, **Figures 2A,C** demonstrate a significant main effect of valproate treatment on protein levels of HDAC1, [*F*(1,24) = 28.99, *p* = 0.0001], and HDAC5, [*F*(1,24) = 11.58, *p* = 0.002], in the male frontal cortex. For HDAC2 protein, significant main effects for DAE, [*F*(1,24) = 10.17, *p* = 0.004], valproate treatment, [*F*(1,24) = 9.464, *p* = 0.005], and a significant interaction between the two, [*F*(1,24) = 13.565, *p* = 0.001], were found in the male frontal cortex, **Figure 2C**. Exposure to arsenic during development significantly increased HDAC1 (*p* = 0.03), HDAC2 (*p* = 0.0004), and HDAC5 (*p* = 0.04) levels in the male frontal cortex after behavioral acquisition and reversal training, as shown in **Figures 2A,C,E**. Treatment with valproate, an HDAC inhibitor, reduced levels of HDAC1 (*p* = 0.0002), HDAC2 (*p* = 0.0004), and HDAC5 (*p* = 0.0135) in the arsenic-exposed male frontal cortex. Thus, changes in HDAC levels induced by DAE in the male frontal cortex were abrogated by treatment with valproate. No effects of DAE nor valproate treatment were observed for HDAC protein levels in the female frontal cortex, **Figures 2B,D,F**. Representative western blots are provided in **Supplementary Figure S1**. Glyceraldehyde-3-phosphate dehydrogenase (GAPDH) is commonly used to evaluate cytosolic contamination of subcellular fractions (Baghirova et al., 2015). Thus, in order to assess the quality of the nuclear preparation that we used in the present studies, we measured levels of GAPDH in 1 ug of nuclear (Gottlicher et al., 2001) fraction and compared that with 1 ug of the post nuclear lysate (PNL, **Supplementary Figure S2**). Quantified levels of anti-GAPDH immunoreactivity were 13.8 times more concentrated in the post nuclear lysate compared to those in the nuclear fraction (intensity units of 1100 in the nuclear band versus 15200 in the post nuclear lysate band), indicating that the nuclear fraction in our preparation is not highly contaminated with cytosolic material. The quantified anti-GAPDH immunoreactivity present in the nuclear preparation may represent GAPDH that is associated with the nucleus (Tristan et al., 2011), rather than cytosolic contamination.

### DAE Increases Bdnf mRNA Expression After Behavioral Training in Males

**FIGURE 3:**
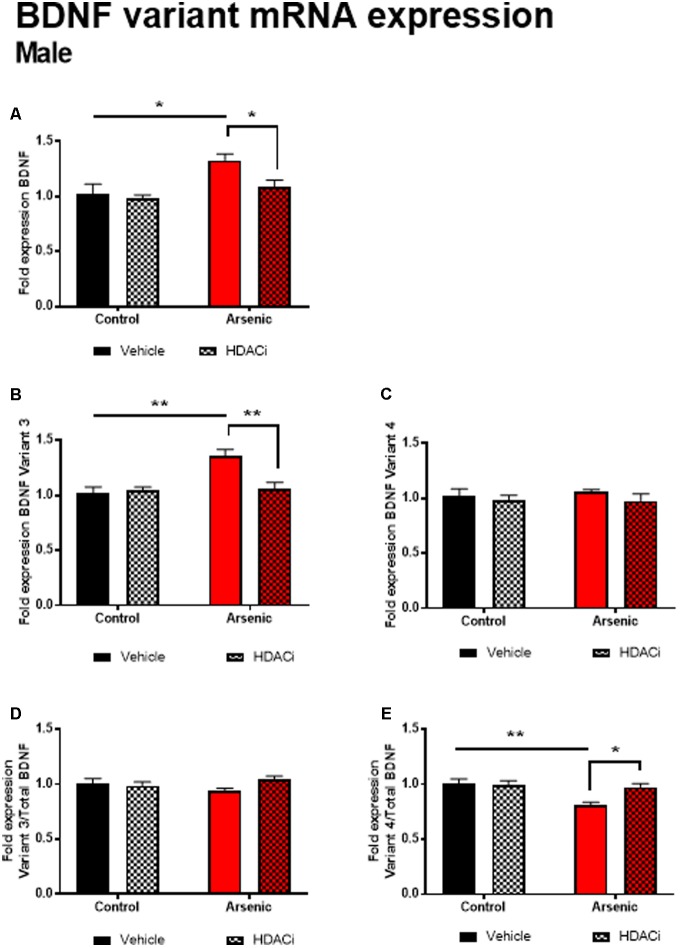
Levels of total BDNF **(A)**, BDNF Variant 3 **(B)**, BDNF Variant 4 **(C)**, BDNF Variant 3/total BDNF **(D)** and BDNF Variant 4/total BDNF **(E)** mRNA from frontal cortex in control (black bars) and arsenic-exposed (red bars) male mice treated with vehicle (solid bars) or HDACi (valproate, stippled bars). Data are mean ± SEM, *n* = 6–7 of mRNA fold expression expressed relative to control vehicle condition. ^∗^*p* < 0.05, ^∗∗^*p* < 0.001. Statistical details are provided in the “Results” section.

**FIGURE 4:**
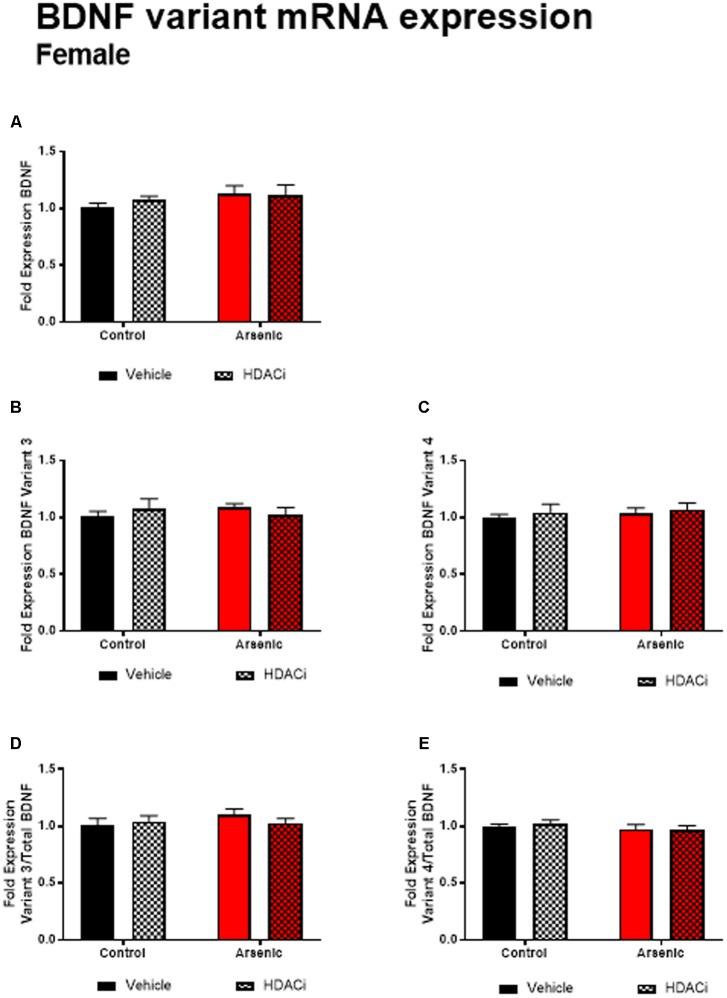
Levels of total BDNF **(A)**, BDNF Variant 3 **(B)**, BDNF Variant 4 **(C)**, BDNF Variant 3/total BDNF **(D)** and BDNF Variant 4/total BDNF **(E)** mRNA from frontal cortex in control (black bars) and arsenic-exposed (red bars) female mice treated with vehicle (solid bars) or HDACi (valproate, stippled bars). Data are mean ± SEM, *n* = 6–7 of mRNA fold expression expressed relative to control vehicle condition. Statistical details are provided in the “Results” section.

Previous research has demonstrated a significant effect of BDNF expression on reversal performance and cognitive flexibility (McCarthy et al., 2016a; Fernandez et al., 2017). We determined mRNA expression of total Bdnf, Bdnf variant 3 (exon IV), and BDNF variant 4 (exon VI) in the frontal cortex of all animals after behavior assessment in the Y-maze. Tissue was prepared for analysis 16 h following achieving Y-maze reversal performance criteria. Tissue from males and females was analyzed using separate qPCR assays; thus, separate two-way ANOVA analysis was utilized to determine the effects of developmental exposure and valproate treatment on BDNF expression within sex. In males, a significant main effect of DAE was determined for total Bdnf mRNA expression, *F*(1,20) = 9.7, *p* = 0.005, Bdnf v3 expression corrected to total Bdnf, *F*(1,20) = 7.4, *p* = 0.013, and Bdnf v4 expression corrected to total Bdnf, *F*(1,20) = 4.3, *p* = 0.009. The same pattern was observed for valproate treatment on Bdnf expression in the male frontal cortex. A significant main effect of valproate treatment was determined for total Bdnf mRNA expression, [*F*(1,20) = 4.5, *p* = 0.05] and for Bdnf v3 expression corrected to total Bdnf, [*F*(1,20) = 4.5, *p* = 0.05]. Significant interactions between DAE and valproate treatment conditions were determined for Bdnf v3, *F*(1,20) = 7.4, *p* = 0.013, and Bdnf v4 corrected to total Bdnf, *F*(1,20) = 5.67, *p* = 0.027. Total Bdnf mRNA expression was elevated in frontal cortex tissue from arsenic-exposed, vehicle-treated males compared to control counterparts (no arsenic exposure), *p* = 0.02, **Figure 3A**. The same pattern was observed for Bdnf v3 expression (*p* = 0.003) in arsenic-exposed male frontal cortex tissue, **Figure 3B**; valproate treatment normalized Bdnf v3 expression similar to control counterparts (no arsenic exposure), but this was not seen when variant 3 is corrected to control (**Figure 3D**). While there were no significant main effects of DAE on Bdnf variant 4, a significant decrease in its expression (*p* = 0.003) was determined when data were presented as the ratio of Bdnf v4 to total Bdnf, **Figure 3E**, but this effect was not present when variant 4 was not corrected to total Bdnf (**Figure 3C**). As observed for HDAC protein expression, no effects of DAE, valproate treatment, and no interaction was found for total Bdnf or Bdnf variant mRNA expression in frontal cortex tissue from female animals (**Figure 4**).

### Valproate Increases Glutamate Receptor Subunit Expression Only in the Female Frontal Cortex

**FIGURE 5:**
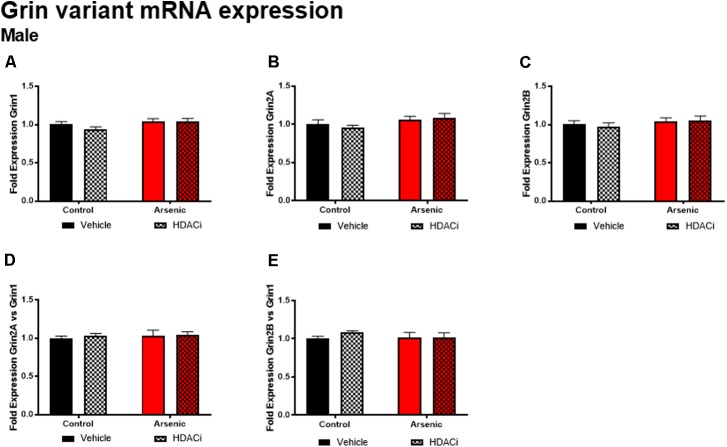
Levels of Grin1 **(A)**, Grin 2A **(B)**, Grin 2B **(C)**, Grin 2A/Grin 1 **(D)**, and Grin 2B/Grin 1 **(E)** mRNA from frontal cortex in control (black bars) and arsenic -exposed (red bars) male mice treated with vehicle (solid bars) or HDACi (valproate stippled bars). Data are mean ± SEM, *n* = 6 of mRNA fold expression expressed relative to control vehicle condition. Statistical details are provided in the “Results” section.

**FIGURE 6:**
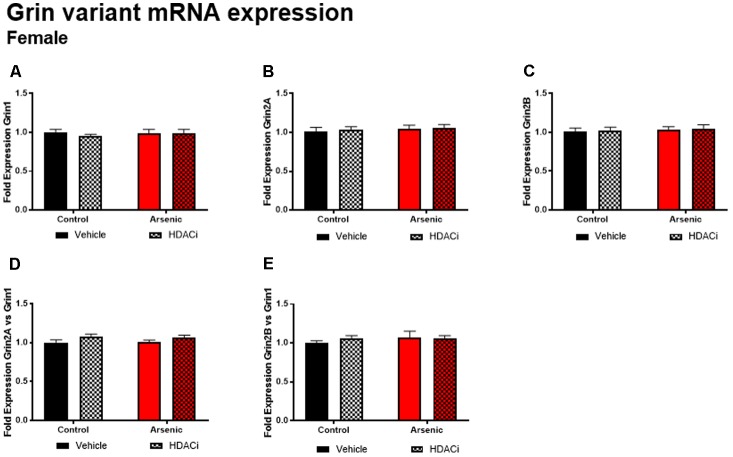
Levels of Grin1 **(A)**, Grin 2A **(B)**, Grin 2B **(C)**, Grin 2A/Grin 1 **(D)** and Grin 2B/Grin 1 **(E)** mRNA from frontal cortex in control (black bars) and arsenic-exposed (red bars) female mice treated with vehicle (solid bars) or HDACi (valproate, stippled bars). Data are mean ± SEM, *n* = 6 of mRNA fold expression expressed relative to control vehicle condition. Statistical details are provided in the “Results” section.

The glutamate receptor, GluN2B, plays a significant role executive function; cortical deletion of Grin2b, the gene for GluN2B, impairs both choice learning and shift choice (reversal) behavior (Brigman et al., 2013). To determine the potential contribution of glutamate receptor subunit expression on reversal learning, we measured Grin1, Grin2a, and Grin2b mRNA expression levels in the frontal cortex of all subjects after Y-maze acquisition and reversal learning. For males, there were no significant effects of DAE, valproate treatment, and no interactions between the two conditions for Grin variant mRNA levels in male frontal cortex tissue (**Figure 5**); indeed, no effect was observed for mRNA expression of Grin2a and Grin2b presented as ratios to Grin1 (**Figure 6**). A similar pattern was observed for female animals: no significant effects of DAE, valproate treatment, and no interactions between the two conditions were found for Grin1, Grin2a, or Grin2b expression. However, a significant effect of valproate treatment was found for Grin2a expression relative to Grin1 expression, *F*(1,20) = 4.993, *p* = 0.037, in the female frontal cortex (**Figure 6D**).

## Discussion

The research investigating arsenic exposure and its role in the development of cancer, cardiovascular disease, psychiatric disorders, and impaired cognition continues to expand. Many studies, using both model organisms and humans, have determined correlations between behavioral outcomes and altered molecular signatures in different brain regions in response to arsenic exposure (Tolins et al., 2014; Tyler and Allan, 2014; Tyler et al., 2014). However, the effect of arsenic exposure on cognitive inflexibility, a behavioral trait observed not only in neurodevelopmental disorders but in neurodegenerative conditions (Bissonette and Powell, 2012), has not been reported. We have demonstrated arsenic’s impact on development of the brain, including effects on molecular pathways controlling neural stem cell differentiation that may propagate into aberrant hippocampal functioning in adulthood. We have proposed that the toxic mechanism of action of this heavy metal, particularly in the brain and among the sexes, is initiated via altered epigenetic processes (Allan et al., 2015; Tyler et al., 2015b, 2017) and thus has the potential to be corrected using epigenetic modifiers. In the present study, we tested the hypothesis that aberrant executive functioning induced by DAE is ameliorated by administration of an epigenetic modifier and that these deficits are apparent in male but not female mice.

Executive functioning refers to a collection of cognitive processes that include reasoning, planning, cognitive flexibility, response inhibition, and sequential and abstract concept formulation (Moreira et al., 2017). Assessment of this functionality in rodents is limited to focusing on cognitive flexibility and response inhibition using either attentional-set shifting or reversal learning tasks. These tasks assess the performance of the dorsolateral and orbital prefrontal cortex, both necessary for discrimination of similar items for successful acquisition and shifting between different stimuli. While reversal learning is less complex than attentional set shifting, it is the preeminent standard for assessment of cognitive flexibility, requiring initial associations between cues and reward and then reversal of those associations with a new reward contingency (Izquierdo and Jentsch, 2012; Izquierdo et al., 2017). Studies have demonstrated that damage to the orbitofrontal cortex leads to normal acquisition of a rewarding behavior but impairs reversal learning of this behavior; thus, orbitofrontal cortical function is a proxy for cognitive flexibility and decision-making skills (Bissonette et al., 2008). We utilized the Y-maze behavioral assay for task acquisition and reversal learning for adult animals exposed to 50 ppb arsenic in drinking water during the perinatal period of development (DAE paradigm). Initial acquisition of the Y-maze task was impaired only in DAE male mice; control male mice and all female mice acquired the initial association to the same degree, with similar trials to criterion. Treatment with valproate, a common medication used for epilepsy, bipolar disorder and depression, which also inhibits HDAC activity, did not significantly improve acquisition performance of the DAE male mice. Conversely, cognitive flexibility, as assessed via reversal learning in the Y-maze, was significantly impaired in DAE male mice and treatment with valproate improved their reversal performance. As observed with acquisition behavior, neither arsenic exposure nor valproate treatment significantly affected female performance in reversal learning, implying resilience of frontal cortical function in females even in the presence of this toxin. These findings suggest that arsenic preferentially affects cognitive flexibility in males only and that addition of valproate, while not improving initial acquisition, does improve performance in reversal learning.

To determine the potential molecular underpinnings of these arsenic-induced behavioral abnormalities, we assessed mRNA and protein levels of *Bdnf* variants and histone deacetylase enzymes, respectively, in the frontal cortex. As observe with behavioral assessments, a significant difference among the sexes was found not only in response to arsenic, but also after valproate treatment. DAE preferentially increased histone deacetylase protein levels (HDAC1, 2, and 5) and levels of total *Bdnf* and its variants in the male frontal cortex. Valproate treatment mitigated and normalized the levels of these proteins and *Bdnf*, reducing levels to normal control levels. None of these changes, induced by either arsenic exposure or valproate treatment were found in the female frontal cortex. Our lab, among others, has previously reported that the female frontal cortex may be more protected from the effects of some toxins (Faulk et al., 2013; Kundakovic et al., 2013; Tyler et al., 2014). Sex-dependent differences in response to arsenic have been well documented; results presented here provide further evidence that females may have an unidentified protective mechanism against arsenic toxicity, particularly during development (Tyler and Allan, 2014; Tyler et al., 2014; Allan et al., 2015). In our previous work, we have identified a potential compensatory mechanism unique to females during embryonic development involving increased glutathione in response to arsenic exposure (Allan et al., 2015). However, we must note that arsenic may affect the adult female brain via an alternative behavioral phenotype that was not measured here (or in other published studies) and may still occur via a currently unknown mechanism. Further study is necessary to elucidate the extent of arsenic toxicity in the female brain and the potential detoxification or compensatory mechanisms that female physiology likely employs to mitigate the effects of arsenic exposure.

We have previously shown that DAE produces increased depressive-like behavior and dysregulation of stress responding in adult males (Martinez et al., 2008; Tyler et al., 2014; Allan et al., 2015; Caldwell et al., 2015). In this present study, a continued elevation of HDAC5, and possibly HDAC1, in the frontal cortex of the arsenic-exposed male mice could indicate chronic stress associated with the reversal-learning task; thus, elevated HDAC levels and stress may induce poor performance and lack of adaptation to the behavioral tasks. Daily treatment with valproate may have mitigated this effect, thereby restoring appropriate behavior in treated subjects for the Y-maze task, but only in male mice. We have previously found decreased levels of H3K9 acetylation (H3K9ac) in the frontal cortex of arsenic-exposed male, but not female, mice; decreased H3K9ac was associated with correspondingly decreased levels of the histone acetyltransferases GCN5 and PCAF, again in male but not female arsenic-exposed mice (Tyler et al., 2015a). Thus, reduced histone acetylation measured under basal conditions support the observation of poorer cognitive flexibility observed in the male arsenic-exposed mice in the present study. This correlates with higher levels of HDAC1 and 5, further exacerbating decreased histone acetylation resulting in poorer cognitive performance as we observed. For female mice, acquisition and reversal learning were not impacted by arsenic and correspond to normal levels of HDAC1 and HDAC5 (measured in the present study) and normal levels of frontal cortical H3K9ac (Tyler et al., 2015a). Finally, HDAC5 is required for associative behavior and is associated with stress susceptibly particularly in the frontal cortex. Overexpression of miR-124 which directly targets HDAC5 (and HDAC4), increases resilience to stress; we have previously found decreased miR-124 in the male embryonic brain with no change in the female brain. Thus, this may allow for overexpression of HDAC5, and via its association with stress and depression, should repress *Bdnf* promoter IV activity. Thus, it is counterintuitive that we found both higher levels of HDAC5 and higher levels of BDNF total and BDNF variant 3 (promoter or exon IV) in the arsenic-exposed male frontal cortex. However, when the data were expressed as the BDNF variant/total BDNF there was a slight reduction for variant 3 and a significant reduction in variant 4. Increased levels of BDNF are generally seen as beneficial to learning; potentially, developmental changes in Bdnf expression are different from elevations, which are activity dependent. Indeed, a recent report suggest that reversal learning deficits due to prenatal cocaine exposure are concurrent with increased BDNF expression and phosphorylated TrkB receptor expression. Selective inhibition of BDNF activation of TrkB restored reversal learning this model of developmental drug exposure (McCarthy et al., 2016b). Although this study indirectly assessed BDNF by blocking the activation of the receptor, results do suggest that elevated frontal cortical BDNF activity can hinder reversal learning performance.

Valproate selectively inhibits the catalytic activity of classes I and II HDACs (Gottlicher et al., 2001) responsible for acetylation of a number of both nuclear and cytoplasmic proteins; thus, pleiotropic effects are possible. HDAC enzymes play a role in associative and spatial memory. Inhibition of specific HDAC enzymes, and the resulting increase in lysine acetylation is therapeutic for cognitive disorders like Alzheimer’s disease (AD) and enhances cognitive performance in rodent models (Swank and Sweatt, 2001; Korzus et al., 2004; Levenson and Sweatt, 2005; Guan et al., 2009; Xu et al., 2011; Yang et al., 2017). Further, specific HDAC enzymes may underlie specific types of learning: deacetylation of histone 3 lysine 9 and overexpression of HDAC1 are both associated with increased fear memory extinction (Bahari-Javan et al., 2012). Spatial memory is associated with HDAC4 (Kim et al., 2012), consolidation of memory is associated with HDAC5 (Agis-Balboa et al., 2013), and knockout of HDAC6 results in restoration of associative and spatial memory in AD models (Govindarajan et al., 2013). In all of these studies, excessive or prolonged expression of HDAC enzymes is detrimental to cognitive performance; thus, while associative, our main finding that arsenic exposure increases HDAC1, 2, and 5 in the male frontal cortex is likely a common underlying mechanism by which cognitive flexibility in adult arsenic-exposed males is impeded.

Preferential response to valproate has been previously reported: valproate administration has a significant effect on male, but not female, performance in some behavioral paradigms (Burenkova et al., 2013). Further, while valproate has been shown to have neuroprotective effects in models of neurodegeneration, these effects are more substantial in males than in females (Long et al., 2016). Valproate administration during development induces autism-like behaviors that are much more pronounced in male than female offspring (Anshu et al., 2017). As we have previously observed for our developmental exposure paradigm, the extent of arsenic toxicity is more severe in altering behavior in exposed males than females (Martinez et al., 2008). Treatment with an epigenetic modifier, like valproate, aimed at increasing acetylation on genes required for learning and memory may be dependent upon the presence of a DAE-induced deficit. This is further supported by the interesting result that valproate treatment does not improve learning in the DAE females; however, arsenic did not impair female performance in reversal learning; thus, the potential for enhanced performance, given the nature of the behavioral assay is limited if a deficit is lacking. Many studies have demonstrated that treatment with valproate enhances learning and memory in a variety of tasks and between both sexes; however, many of these tasks are based on hippocampal, not frontal cortical, functioning. Thus, lack of enhanced cognitive flexibility in the females (both DAE and control) could be due to lack of behavioral deficit, valproate dosing, or some unidentified molecular mechanism that we have yet to ascertain.

Several limitations in the present study merit mention. First, we did not assess mRNA levels of HDACs. Therefore, we do not know whether the observed changes in protein levels were the result of altered expression (transcription) or, alternatively, protein turnover. Second, due to the limited tissue available, we only measured mRNA transcript for BDNF and the glutamate receptors. Thus, we are not able to draw conclusions about the effects of either prenatal arsenic or the valproate treatments on protein. The changes we have observed both in HDAC protein and BDNF mRNA expression should be followed-up with future studies.

## Conclusion

We have shown that DAE is associated with impaired acquisition and reversal learning in male, but not female, mice. Valproate treatment corrected the deficit in reversal learning, while not affecting the deficit in initial acquisition of Y-maze learning for male mice. Further, measures of HDAC1, 2 and 5 levels obtained following the reversal learning phase of the test revealed that DAE was associated with increased levels of all three HDACs for which treatment with valproate reversed. Neither DAE nor valproate treatment altered behavior or HDAC levels in female mice. However, DAE and valproate treatment concurrently altered total Bdnf and Bdnf variant 3 and variant 4 mRNA levels in males with compromised behavioral performance. Research described herein identifies a sex-dependent effect of DAE on cognitive flexibility and a potential epigenetic mechanism of action underlie treatment of these deficits. The resiliency of females to the damaging actions of DAE is an area of research that warrants further investigation; however, we can confirm that treatment with valproate will improve frontal cortical function in adult males exposed to an environmental toxin during development. Such a finding will help inform potential mitigation and treatment efforts for arsenic-induced cognitive deficits for those individuals exposed during childhood.

## Ethics Statement

All experiments were carried out under a University of New Mexico HSC IACUC approved animal protocol (15-200383-HSC). All personnel were approved for the work and complied with all training requirements. The HSC-Animal Facility is accredited and holds both an Animal Welfare Assurance (#D16-0028 (A3350-01) and USDA Registration (#85-R-0014).

## Author Contributions

CT conceived of the HDACi experiments and helped with the research design. She produced the arsenic exposed mice used in the studies and helped to write the manuscript. JS was the technician performing the Grin and BDNF mRNA analysis. ES was responsible for the detailed analysis of the nuclear fractions and quantification of the housekeeping genes as well as HDAC immunoblots. EV was responsible for demonstrating the purity of the nuclear preparation and completing the HDAC immunoblotting. KC developed the primers and identified the antibodies used. He developed the protocol for the immunoblotting and mRNA work and helped to write the manuscript. AA ran the behavioral studies, performed the daily HDACi injections, dissected the tissue, conducted the statistical analyses, wrote the manuscript and her lab provided the funding for the project through her NIH grant. All authors analyzing the data, writing of methods and results as well as editing the discussion section.

## Conflict of Interest Statement

The authors declare that the research was conducted in the absence of any commercial or financial relationships that could be construed as a potential conflict of interest.

## Supplementary Material

FIGURE S1 Representative western blots for HDAC assessment in male and female frontal cortex with and without valproate treatment.

FIGURE S2 Representative western blot probed for GAPDH in 1 μg of loaded protein from nuclear (NUC) and post nuclear lysate (PNL) fractions of mouse frontal cortex.
