# Supplementary figures and images for: Sex-Dependent Effects of the Histone Deacetylase Inhibitor, Sodium Valproate, on Reversal Learning After Developmental Arsenic Exposure
Source: Front Genet. 2018 Jun 15;9:200. doi: 10.3389/fgene.2018.00200 (PMC6013562; doi:10.3389/fgene.2018.00200)

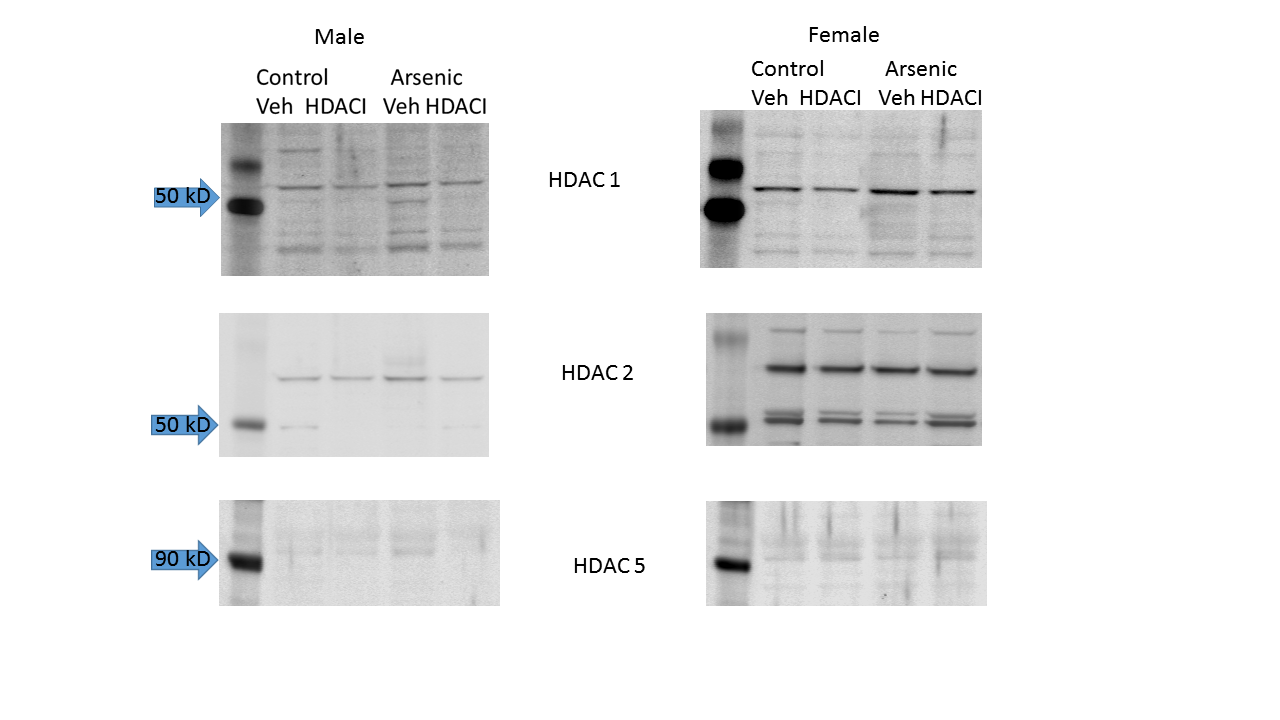

Supplement: FIGURE S1 — Representative western blots for HDAC assessment in male and female frontal cortex with and without valproate treatment. [file Image_1.tif]

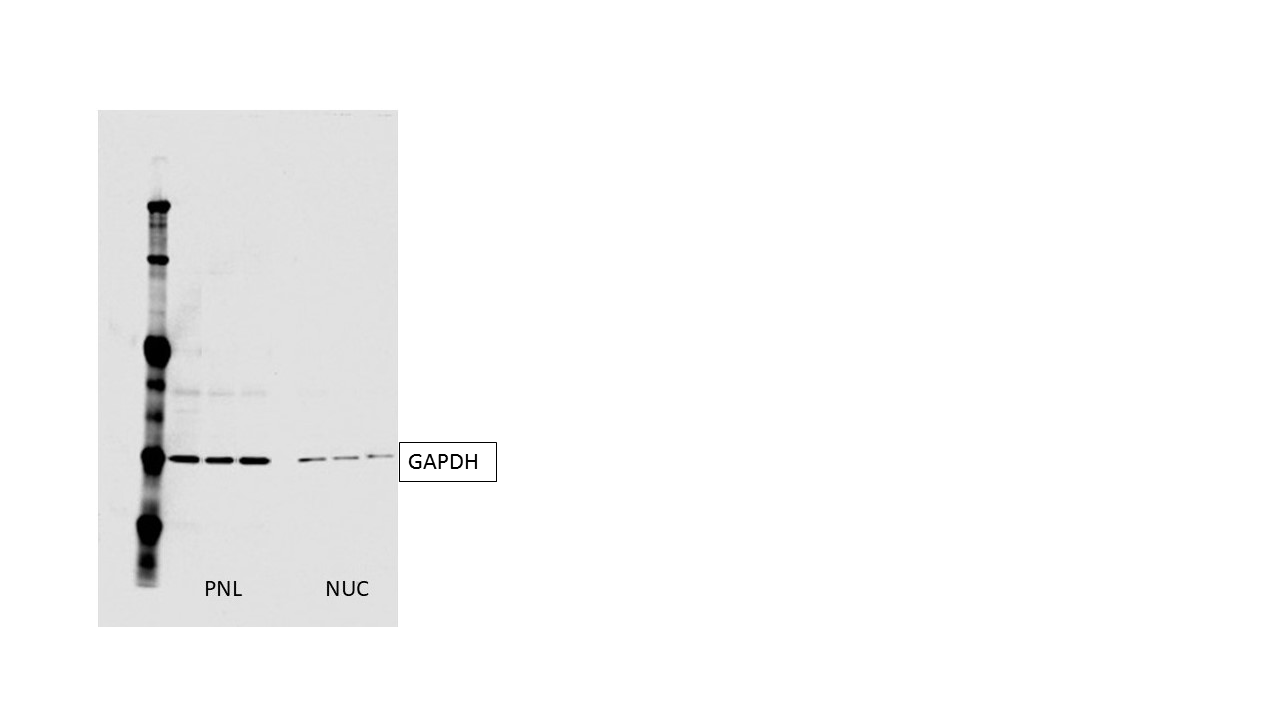

Supplement: FIGURE S2 — Representative western blot probed for GAPDH in 1 μg of loaded protein from nuclear (NUC) and post nuclear lysate (PNL) fractions of mouse frontal cortex. [file Image_2.tif]
